# Compact steady-state tokamak performance dependence on magnet and core physics limits

**DOI:** 10.1098/rsta.2017.0440

**Published:** 2019-02-04

**Authors:** J. E. Menard

**Affiliations:** Princeton Plasma Physics Laboratory, Princeton, NJ, USA

**Keywords:** magnetic fusion, tokamak, high temperature superconductors, pilot plant

## Abstract

Compact tokamak fusion reactors using advanced high-temperature superconducting magnets for the toroidal field coils have received considerable recent attention due to the promise of more compact devices and more economical fusion energy development. Facilities with combined fusion nuclear science and Pilot Plant missions to provide both the nuclear environment needed to develop fusion materials and components while also potentially achieving sufficient fusion performance to generate modest net electrical power are considered. The performance of the tokamak fusion system is assessed using a range of core physics and toroidal field magnet performance constraints to better understand which parameters most strongly influence the achievable fusion performance.

This article is part of a discussion meeting issue ‘Fusion energy using tokamaks: can development be accelerated?’.

## 1. Introduction

A fusion nuclear science facility (FNSF) is viewed as a potentially important element of the U.S. fusion development roadmap that would be complementary to the DEMO-based approach pursued by the majority of the world fusion program. In particular, a FNSF/ component test facility (CTF) [1–7] could make significant contributions to the development of fusion energy by providing the nuclear environment needed to develop fusion materials and components. The FNSF nuclear environment includes fusion-relevant neutron wall loading *W*_*n*_≥1 MW m^−2^, neutron fluence greater than or equal to 6 MW yr m^−2^, component testing area of 5–10 m^2^ and continuous on-time (i.e. steady-state operation) for durations in the range of 10^6^ s [8]. Materials testing, blanket testing, and demonstrating predictable tritium breeding and T self-sufficiency commonly define critical elements of the FNSF mission [9]. A Pilot Plant [10–12] would be a device capable of performing the FNSF/CTF mission while also incorporating features including actuators with high wall-plug efficiency and low power consumption and breeding blankets with high thermal conversion efficiency, all to support the production of modest net electricity (50–300 MWe). Maintenance schemes applicable to a power plant including methods for rapid replacement of in-vessel components would also be a key feature of a Pilot Plant.

The required lifetime of fusion facility components and infrastructure is directly linked to the mission(s) of the facility. The ‘FNSF-Pilot’ facility approach described here aims to be directly scalable to a power plant in a device as small as possible to reduce device cost and risk. Such an approach may result in reduced shielding and breeding blanket thickness relative to larger DEMO devices [13–15] which aim to operate at higher fusion power and neutron fluence. Thus, one challenge of the FNSF-Pilot approach may be reduced magnet and/or facility lifetime, and this must be balanced against the potential benefits of reduced device size and cost. Previous studies have explored the impact of varied aspect ratio on compact tokamak devices with a combined FNSF and Pilot Plant mission. In particular, high-temperature superconducting (HTS) toroidal field (TF) coils offer the potential to enable the achievement of high fusion gain and power in smaller major radius devices [16–19]. The work described here further explores the role of magnet engineering limits and plasma core confinement and stability limits on fusion performance with varied aspect ratio. The configurations studied are direct extensions of the HTS-TF FNSF-Pilot Plants studied in [17] with *R* = 3 m, 50 MW of 0.5 MeV Negative Neutral Beam Injection (NNBI) heating and current drive, Greenwald density fraction of 0.8 and sufficient shielding to protect the HTS magnet for several full-power years (baseline scenario) and sufficient breeding for tritium self-sufficiency. The remainder of this paper is outlined as follows: Section 2 describes compact tokamak fusion performance scalings versus normalized beta, elongation, magnet parameters and confinement assumptions, Section 3 summarizes with conclusions and appendix A provides more detail on the scaling of the fusion gain with dimensional and dimensionless parameters relevant to steady-state tokamak configuration optimization.

## 2. Compact tokamak fusion performance scalings

The results described here are a direct extension of the 0D systems-code methodology used in §5 of [17]. The assumed normalized beta *β*_*N*_, elongation *κ* and vacuum toroidal magnetic field at the plasma geometric centre *B*_*T*_ dependence on aspect ratio plays a very strong (approx. quartic) role in the projected fusion performance of high-bootstrap-fraction tokamak scenarios since *P*_fusion_∝*ϵ*(*β*_*N*_*κB*_*T*_)^4^ [17]. This scaling shows that the achievable *β*_*N*_, *κ* and *B*_*T*_ influence the fusion power on a similar footing for steady-state tokamak scenarios. Furthermore, the inboard blanket and shield thickness assumptions play an important role in determining the space available for the inboard toroidal field magnet and associated support structure as described in the following subsection. In subsequent subsections, the plasma stability, elongation, magnet parameters and confinement scalings are varied to develop an understanding of which parameters most strongly influence fusion performance for *R*=3m pilot plants with 50 MW of 0.5 MeV deuterium NNBI auxiliary heating and current drive. Compact tokamak devices in this size and power range are projected to be capable of achieving net electricity production in steady-state if the toroidal magnetic field, energy confinement and MHD stability limits are sufficiently high.

### (a) Model for shielding and tritium breeding blanket thickness

**Figure 1.**
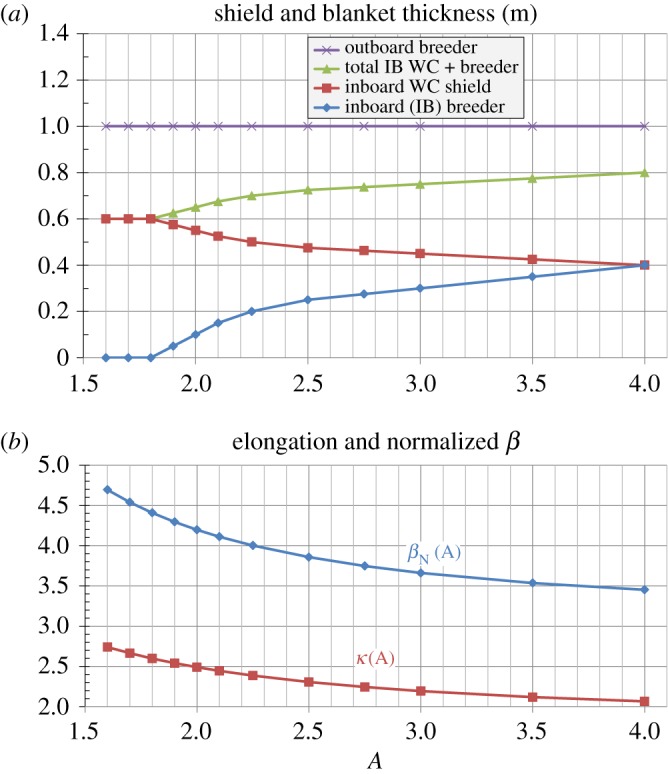
(*a*) Inboard breeding blanket and shield thickness and outboard breeding blanket thickness versus aspect ratio *A*, (*b*) normalized beta *β*_*N*_(*A*) and double-null plasma boundary elongation *κ*(*A*). (Online version in colour.)

Figure 1*a* shows the inboard breeding blanket and shield thickness and outboard breeding blanket thickness versus aspect ratio *A* used in this study and previous HTS-TF pilot-plant study [17]. Both inboard and outboard breeding blankets are assumed to be dual-cooled lead lithium (DCLL) [20]. First, for sufficiently low *A* (*A* = 1.7–1.8) and sufficiently large major radius (*R*≥1.7 m) detailed neutronics analysis indicates no inboard breeding blanket is required to achieve TBR ≈ 1 [17,21] as long as breeding is provided at the top and bottom of the device, NBI heating aperture areas are sufficiently small and NBI ducts are tangential to the plasma. For HTS TF magnets, sufficient shielding is required to protect against both nuclear heating and/or neutron damage. Detailed calculations including ferritic steel structure (which has lower neutron attenuation) to support and contain a WC shield and water coolant indicate shield-averaged attenuation decay lengths of 15–16 cm per decade, and this results in a requirement for approximately 60 cm of cooled WC shield (or equivalent shielding) to protect the HTS TF magnet in a device that can achieve a peak outboard neutron fluence of 7 MW yr m^−2^ [22,23]. These results motivate the inboard WC shielding thickness of 60 cm at *A* = 1.8 as shown in figure 1*a*. Furthermore, at *A* = 2, there is insufficient outboard surface area to achieve TBR greater than or equal to 1 without inboard breeding. However, a 10 cm thick DCLL inboard breeding blanket can provide sufficient inboard breeding to achieve TBR greater than or equal to 1. This blanket is approximately half as effective for shielding as 10 cm of WC, so the inner WC shield can be reduced to 55cm thickness for a total shield plus breeding blanket thickness of 65 cm at *A* = 2.

Dedicated neutronics calculations have not been carried out for higher *A* for an HTS-TF-based R=3m FNSF-Pilot. However, one can refer to the previous calculations finding that *A* = 4 advanced Tokamak (AT) pilot plants [12] with 40 cm of inboard breeding blanket (and 80 cm of outboard breeding blanket) can achieve TBR ≥ 1. These results correspond to the inboard shielding and breeding curves in figure 1*a* at *A* = 4 chosen to provide shielding equivalent to 60 cm of WC shielding. The inboard breeding blanket thickness for other aspect ratios is determined via interpolation while matching the aforementioned thickness values at *A* = 1.8, 2.0 and 4. Then, the WC shielding thickness is chosen so that all aspect ratios have an equivalent WC shielding thickness of 60 cm again assuming a DCLL blanket of a given thickness provides the same shielding as a WC shield half as thick. Clearly, the model curves in figure 1*a* are approximations and additional three-dimensional neutronics calculations are required to provide a more accurate dependence on *A*. As a cross-check, it is noted that the *R* = 2.7, *A* = 3.5 fusion development facility (FDF) [24,25] with copper magnets requires a 25 cm thick inboard DCLL blanket to achieve TBR ≥ 1. This implies a 73 cm inboard WC shield plus blanket total thickness is required to achieve a 60 cm WC-only shield equivalent, whereas figure 1*a* indicates 78 cm at *A* = 3.5. This indicates that figure 1*a* captures the leading order trends for required inboard shielding and breeding blanket thickness for TBR ≥1 for the range of aspect ratios considered here. Lastly, it is noted that small changes in inboard shielding thickness can have major impacts on device performance and lifetime. For example, an increase in effective inboard shielding thickness from 60 cm to 70 cm reduces the fusion and engineering gain by 25–35% for *A* = 2–3 but increases the peak outboard neutron fluence by a factor of 4 and increases HTS magnet lifetime by a factor of 5 in full-power-years [17].

### (b) Aspect ratio dependence of normalized beta and elongation

**Figure 2.**
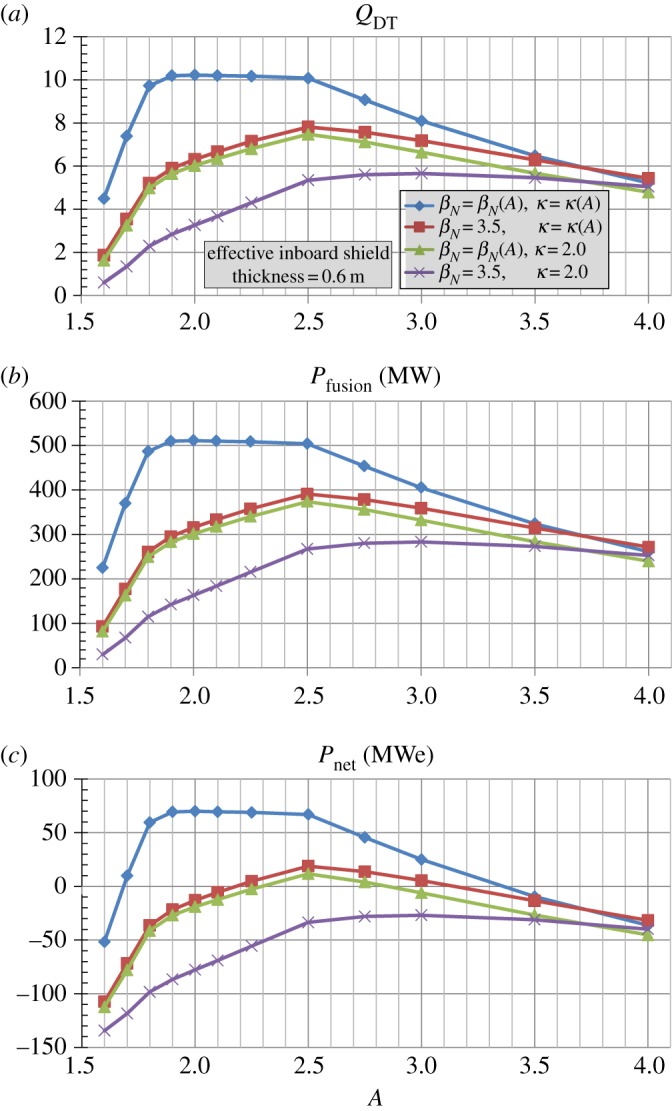
(*a*) Fusion gain *Q*_*DT*_, (*b*) fusion power *P*_fusion_ and (*c*) net electrical power for *R*_0_ = 3 m HTS ST/AT pilot plants with effective inboard shielding thickness = 0.6 m versus aspect ratio *A* for different assumptions for *β*_*N*_(*A*) and *κ*(*A*). (Online version in colour.)

Figure 1*b* shows the assumed maximum *β*_*N*_(*A*) (*n* = 1 no-wall kink stability limit), *κ*(*A*), and shielding and blanket thickness versus aspect ratio *A* used in these studies as was done in [17]. The simplifying assumption is made that the *β*_*N*_(*A*) and *κ*(*A*) scalings are independent of absolute parameters such as device size, field or current and plasma parameters such as collisionality, toroidal rotation speed, poloidal beta or internal inductance. Holding the HTS winding pack thickness fixed at 0.24 m and varying the inboard toroidal field coil structure thickness versus *A* (increasing from 0.2 m to 0.45 m between *A* = 2 and 4) as was also done in [17], figure 2*a* shows the fusion gain *Q*_*DT*_, figure 2*b* the fusion power and figure 2*c* the net electric power versus *A* for an assumed effective inboard WC shield thickness of 0.6 m and varied assumptions of *β*_*N*_(*A*) and *κ*(*A*). As is evident from figure 2, fixing either *β*_*N*_ or *κ* constant (at values of 3.5 and 2.0, respectively) significantly reduces the achievable fusion gain and power at lower aspect ratios and indicates an optimal aspect ratio near *A* = 2.5. Furthermore, holding both *β*_*N*_ = 3.5 and *κ* = 2.0 fixed further reduces the fusion gain and power at low-*A* and makes the fusion gain and power nearly independent of aspect ratio for *A*≥2.5. Given the importance of *β*_*N*_(*A*) and *κ*(*A*) on fusion performance evident in figure 2, determining whether the projected *β*_*N*_ and *κ* values are sustainable in fully non-inductive low-aspect-ratio scenarios is a major research goal for the future NSTX-U operation [26].

### (c) Fusion performance versus toroidal field magnet parameters

**Figure 3.**
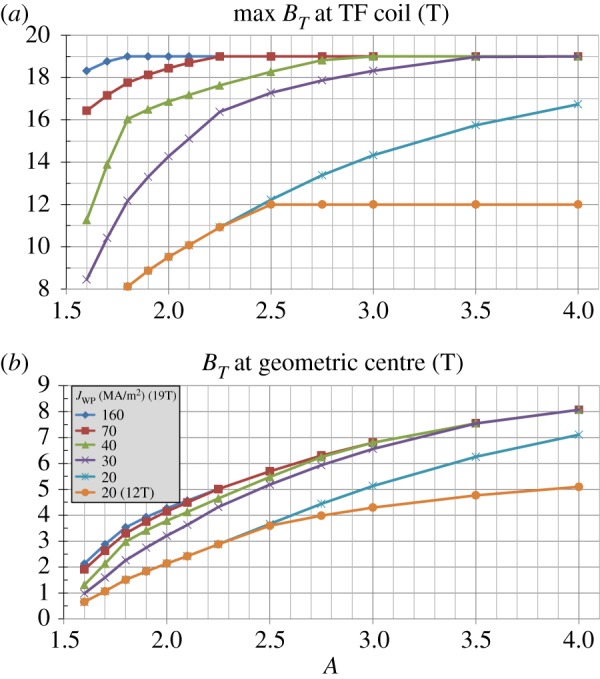
(*a*) Maximum toroidal magnetic field at magnet, (*b*) vacuum toroidal magnetic field at plasma geometric centre versus aspect ratio for various toroidal field coil maximum field and winding pack current density assumptions. (Online version in colour.)

The results of figure 2 are representative of expected fusion performance for a particular choice of toroidal field coil winding pack thickness (0.24 m) and structural support width variation with aspect ratio that provides for sufficient space for at least a small central solenoid for *A*≥2 and results in maximum magnetic field values ranging from 17 T to 19 T at the TF magnet. The results in figure 2 assume a maximum allowable TF structural support stress of 0.66 GPa typical of stainless steel, winding pack current density 70 MAm^−2^, and winding pack stress limited to 0.4 GPa to ensure strains less than or equal to 0.3% to avoid any stress-related degradation in critical current [17]. To better understand the role of advanced superconductors on the achievable fusion performance versus aspect ratio, different combinations of maximum magnetic field at the magnet $B_{\max}$ and winding pack current density are considered as shown in figure 3. An important reference case is the ITER toroidal field coil set [27–29] with $B_{\max}\approx 12\;T$ and *J*_WP_ = 20 MAm^−2^. Figure 3*a* shows that for these magnet parameters the maximum allowable magnetic field cannot be reached for *A* ≤ 2.5 due to space constraints on the inboard TF magnet. Furthermore, as $B_{\max}$ is increased to 19 T [30], higher *J*_WP_ ≈ 30 MAm^−2^ is required to access this higher maximum magnetic field for *A*≥3.5. Progressively higher *J*_WP_ up to 160 MAm^−2^ [31,32] is required to reach $B_{\max}=19\;T$ for nearly all aspect ratios, i.e. for *A*≥1.8. There is only a small or no increase in the accessible magnetic field (assuming $B_{\max}=19\;T$) for *A*≥2 for *J*_WP_≥70 MAm^−2^. Figure 3*b* shows the impact of using ITER LTS magnet assumptions versus advanced high *J*_WP_ REBCO HTS magnet assumptions. In particular, the accessible vacuum toroidal field in the plasma increases by approximately a factor of 3 at *A* = 1.6, a factor of 2 at *A* = 2, a factor of 1.6 for *A*≥2.5.

**Figure 4.**
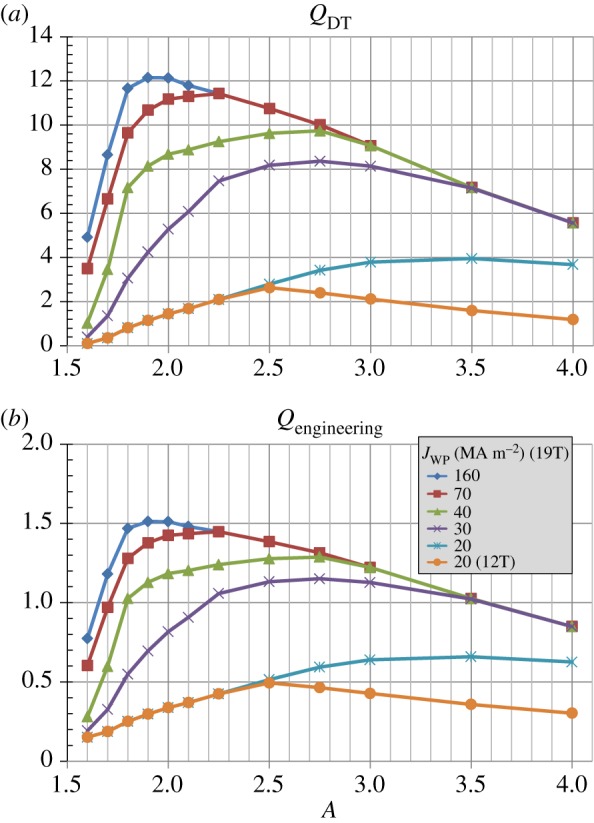
(*a*) Fusion gain, (*b*) engineering gain versus aspect ratio for various toroidal field coil maximum field and winding pack current density assumptions. (Online version in colour.)

**Figure 5.**
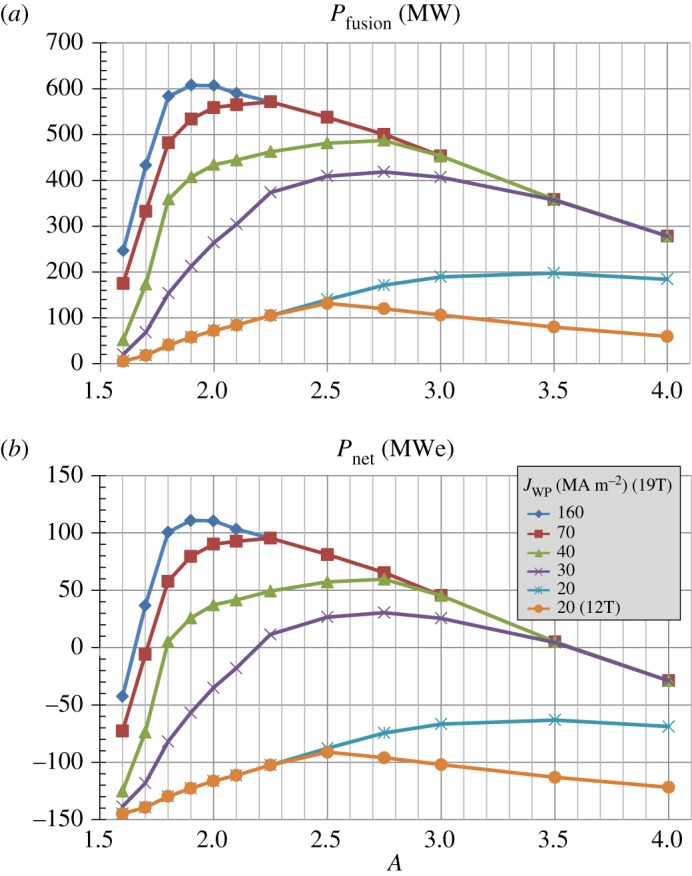
(*a*) Fusion power, (*b*) net electric power versus aspect ratio for various toroidal field coil maximum field and winding pack current density assumptions. (Online version in colour.)

Figure 4 shows that the maximum fusion and electricity gains are sensitive functions of both magnet parameters and aspect ratio. For ITER-like magnet parameters of $B_{\max}\approx 12\;T$ and *J*_WP_ = 20 MAm^−2^, the fusion gain is constrained to be less than 3 while *Q*_eng_ ≤ 0.5. Increasing the maximum field constraint from 12 T to 19 T increases *Q*_*DT*_ to above 4 but *Q*_eng_ remains less than unity. However, for $B_{\max}\approx 19\;T$ and *J*_WP_ = 30 MAm^−2^, the fusion gain increases by a factor of 2 and *Q*_eng_≥1 between *A* = 2.2 and 3.5. As shown in figure 5, as the winding pack current density is further increased the aspect ratio that produces the highest fusion power and net electric power approaches *A* ≈ 2 with *P*_fusion_ up to 600 MW and net electric power greater than 100 MWe. These results highlight the importance of the simultaneous high winding pack current density and high maximum field of HTS to leverage increased normalized plasma stability at reduced aspect ratio for maximizing fusion power and net electric power in a compact tokamak.

**Figure 6.**
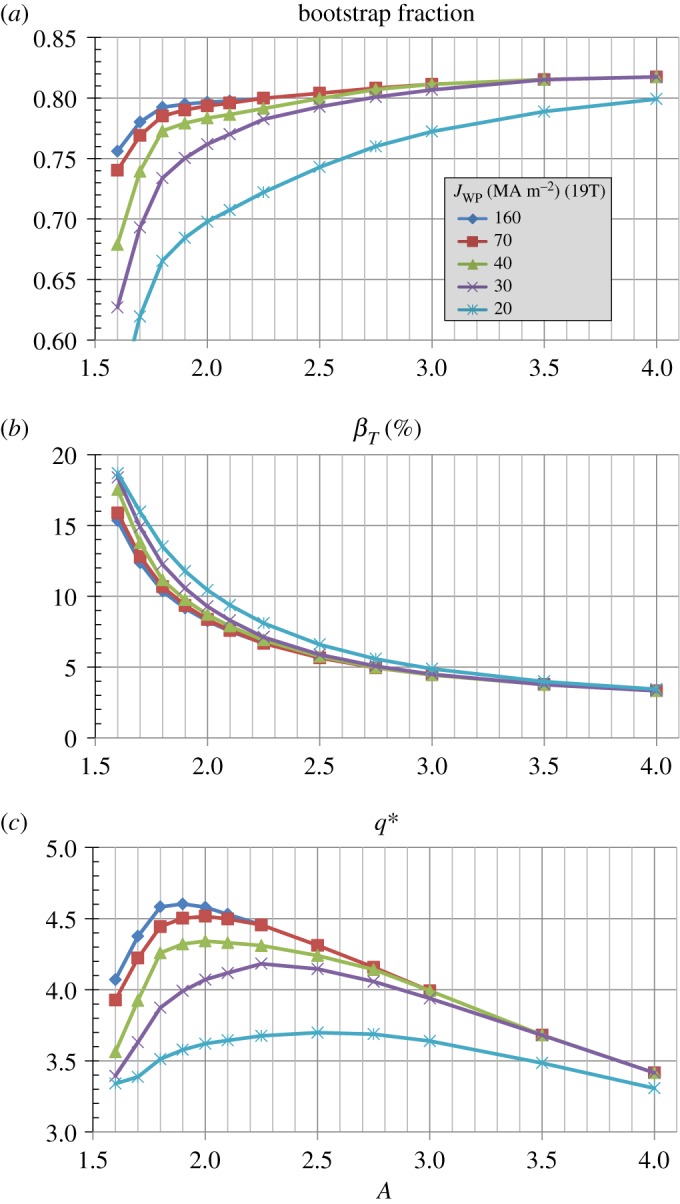
(*a*) Bootstrap current fraction, (*b*) toroidal beta and (*c*) kink safety factor versus aspect ratio for various winding pack current density assumptions at fixed toroidal field coil maximum magnetic field of 19 T. (Online version in colour.)

Figure 6*a* shows that for *J*_WP_≥30 MAm^−2^ nearly all aspect ratios have bootstrap fractions between 70% and 85% with average values near 80% for higher *A*. Figure 6*b* shows that the toroidal beta increases nearly inverse-quadratically with reduced aspect ratio (*β*_*T*_(%) ≈ 36*A*^−1.8^) and increases by roughly a factor of 5 between *A* = 4 and *A* = 1.6. Figure 6*c* shows that the kink safety factor q* is above 3 for all aspect ratios analysed and increases to up to 4.5 near *A* ≈ 2 for the highest winding pack current densities.

All of the results shown in figures 4 through 6 assume the energy confinement is sufficient to operate at the *β*_*N*_ limit shown in figure 1*b*. To better understand the potential challenge in accessing these *β*_*N*_ values in a high-field compact device, the required confinement is compared to several common confinement scalings. The most commonly used scaling is the ITER 98y2 H-mode confinement scaling [33,34] given by the following expression: $\tau_{E-98y2}[s]=0.0562\times I_{P}{\left[MA\right]}^{0.93}B_{T}{\left[T\right]}^{0.15}P{\left[MW\right]}^{-0.69}{\bar{n}}_{e}{\left[10^{19}\;m^{-3}\right]}^{0.41}M^{0.19}R{\left[m\right]}^{1.97}\epsilon^{0.58}\kappa^{0.78}$. In contrast to the ITER scaling which varies as *β*^−0.9^ [34] when expressed in dimensionless parameters, several device-specific confinement scaling studies from JET, DIII-D and NSTX [35] have shown that the confinement scales weakly with *β*. Such weak *β* dependence is consistent with electrostatic turbulence with gyro-Bohm scaling represented in the ‘Petty08’ scaling expressed as: $\tau_{E-Petty08}[s]=0.052\times I_{P}{\left[MA\right]}^{0.75}B_{T}{\left[T\right]}^{0.30}P{\left[MW\right]}^{-0.47}{\bar{n}}_{e}{\left[10^{19}\;m^{-3}\right]}^{0.32}M^{0.0}R{\left[m\right]}^{2.09}\epsilon^{0.84}\kappa^{0.88}$.

There are several ST confinement scalings in the literature [36–39] with variations depending on the parameters included in the fit, the fitting method used, and the machine considered (NSTX versus MAST). The ST confinement scaling assumed here uses the Case 1 ordinary least-squares regression (OLSR) exponents for current, field, density and power from NSTX [36] and assumes ITER 98y2 exponents are applicable where the ST exponents are not yet determined, i.e. for the species mass, major radius, inverse aspect ratio and elongation. As discussed in [36], the OLSR analysis assumes perfect knowledge of both the independent (predictor) variables and the dependent (response) variable with no uncertainties in either the independent or dependent values. The OLSR analysis used here has somewhat lower root mean square error (RMSE) compared to the principal component error-in-variable (PCEIV) technique for fitting to the NSTX data. An ‘NSTX’ ST confinement scaling is then given by the following expression: $\tau_{E-NSTX}[s]=0.095I_{P}{\left[MA\right]}^{0.57}B_{T}{\left[T\right]}^{1.08}P{\left[MW\right]}^{-0.73}{\bar{n}}_{e}{\left[10^{19}\;m^{-3}\right]}^{0.44}M^{0.19}R{\left[m\right]}^{1.97}\epsilon^{0.58}\kappa^{0.78}$. The leading NSTX confinement scaling coefficient is chosen such that the ITER and ST energy confinement times are identical for a reference NSTX scenario defined by *A* = 1.5, *R*_0_ = 0.86 m, *I*_*P*_ = 0.75 MA, *B*_*T*_ = 0.5*T*, *P*_*NBI*_ = 4 MW and *f*_*GW*_ = 1.0 consistent with a total *β*_*N*_ = 4.4 using the 0D scaling methodology outlined in [26]. Other similar ST scaling expressions are of course possible, and obtaining a more definitive ST confinement scaling at reduced collisionality and higher magnetic field and current is a major research goal for both NSTX Upgrade [26,40–43] and MAST Upgrade [44–46].

In addition to the uncertainty in confinement scaling at low aspect ratio there is also uncertainty in how any scaling differences at low aspect ratio transition or connect to higher aspect ratio. Any such transitions/connections between low and higher aspect ratio confinement could have important implications for the ultimate choice of aspect ratio for compact pilot plants. To assess this in a highly approximate way, a hybrid ‘NSTX-Petty08’ scaling is used. This *ad hoc* hybrid scaling accounts for the fact that the NSTX ST scaling has thus far been developed for plasmas with *A* ≤ 1.7 (*ϵ*≥0.6). Similarly, the Petty08 scaling has been developed using data primarily from conventional aspect ratio plasmas with *A*≥2.5 (*ϵ* ≤ 0.4). Assuming a linear interpolation in *ϵ* between the two scalings is justified, a combined/hybrid scaling can be defined as the weighted sum of the NSTX and Petty08 scalings according to: *τ*_*E*_ = *τ*_E−NSTX_ for *ϵ*≥*ϵ*_1_ = 0.6, *τ*_*E*_ = *τ*_E-Petty08_ for *ϵ* ≤ *ϵ*_2_ = 0.4 and *τ*_*E*_ = (*ϵ* − *ϵ*_2_)/(*ϵ*_1_ − *ϵ*_2_)*τ*_E-*NSTX*_ + (*ϵ*_1_ − *ϵ*)/(*ϵ*_1_ − *ϵ*_2_)*τ*_E-Petty08_ for *ϵ*_2_ < *ϵ* < *ϵ*_1_.

**Figure 7.**
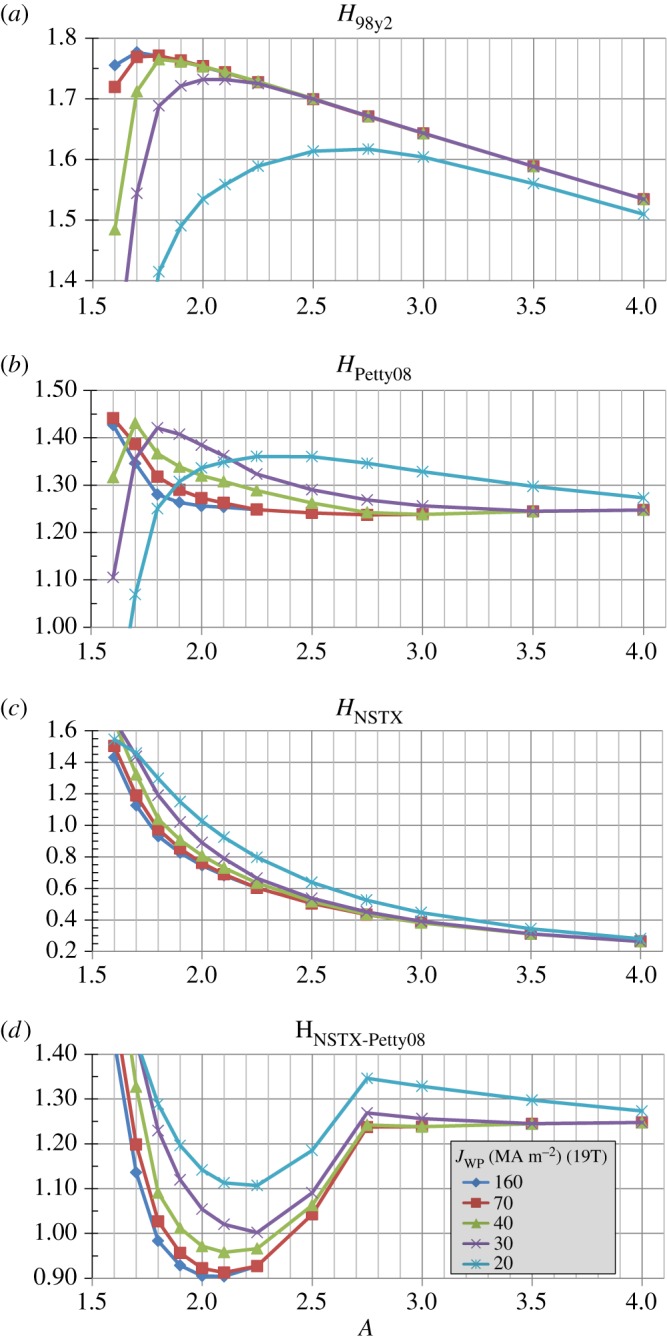
Normalized confinement enhancement factors for (*a*) ITER 98y2, (*b*) Petty08, (*c*) NSTX and (*d*) hybrid NSTX-Petty08 energy confinement scalings versus aspect ratio for various winding pack current density assumptions at fixed toroidal field coil maximum magnetic field of 19 T. (Online version in colour.)

Figure 7*a* shows that in order to reach the *β*_*T*_ values shown in figure 6*b* elevated normalized confinement is required for all aspect ratios. In particular, the required H-factor relative to the ITER 98y2 scaling is as high as 1.7–1.8 at lower *A* and is 1.5–1.6 for high *A* = 3-4. Similarly, figure 7*b* shows that even for the Petty08 confinement scaling with weak *β* dependence, the H-factor relative to the scaling is between 1.2 and 1.4 for nearly all aspect ratios and is nearly independent of *A* with *H*_Petty08_ ≈ 1.25 for *A*≥2 for the highest winding pack current densities. Figure 7*c* shows the NSTX ST confinement scaling H-factor needed to achieve the assumed *β*_*T*_ is a rapidly decreasing function of increasing aspect ratio. Again, this ST scaling has not yet been extended to higher field, current or plasma temperature (i.e. reduced collisionality) and is obviously not applicable to higher aspect ratios *A* > 2.5. Figure 7*d* shows the NSTX-Petty08 confinement scaling H-factor needed to achieve the assumed *β*_*T*_ is near or below 1 for *A* in the range of 1.8 to 2.3 for the higher winding-pack current density cases with *J*_WP_≥40 MAm^−2^. This result opens up the interesting (albeit speculative) possibility of the optimal aspect ratio from an integrated magnet, shielding, confinement, stability and non-inductive sustainment standpoint being between *A* = 1.8 and 2.3—a range of aspect ratios not previously studied experimentally.

**Figure 8.**
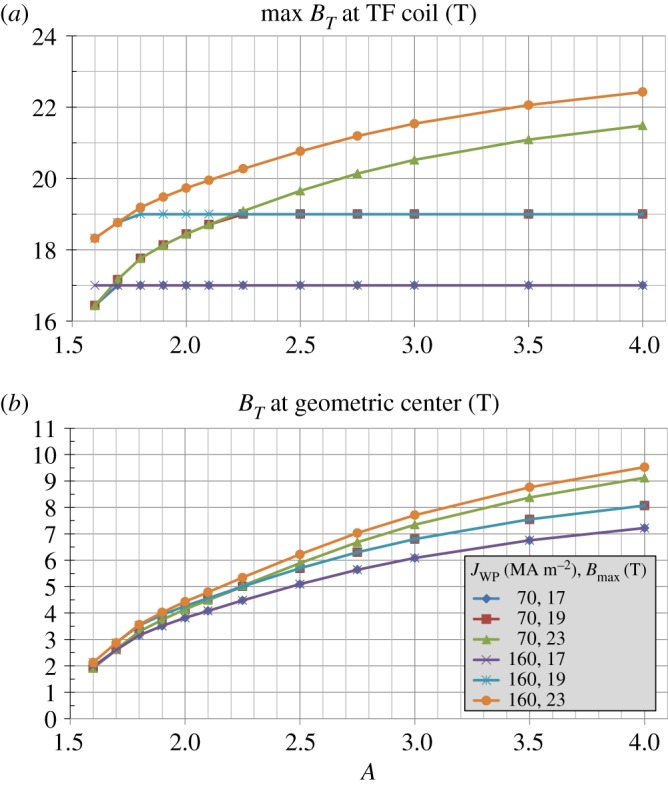
(*a*) Maximum toroidal magnetic field at magnet, (*b*) vacuum toroidal magnetic field at plasma geometric centre versus aspect ratio for various toroidal field coil maximum field and winding pack current density assumptions. (Online version in colour.)

A further consideration in the aspect ratio optimization of compact tokamaks is the impact of the maximum magnetic field achievable or allowed at the magnet. To gain further insight into this dependence, a set of three maximum fields is considered (17, 19 and 23T) in combination with a set of two winding pack current densities (70 and 160 MAm^−2^) consistent with conductor on round core (CORC) magnet cables under development [30–32]. Figure 8 shows the achievable fields for this combination of magnet parameters. Figure 8*a* shows the maximum achievable toroidal magnetic field for these parameters and indicates that nearly all aspect ratios (*A* = 1.6 is the exception) can achieve 17T at the magnet for both *J*_WP_ assumptions. Figure 8*a* also shows that *A*≥2.2 is required to achieve 19 T at the magnet for both *J*_WP_ assumptions and that *A*≥1.8 can achieve 19 T for *J*_WP_ = 160 MAm^−2^. None of the aspect ratios considered here can achieve 23 T at the magnet due to inboard structural space limitations, but *A* = 4 and *J*_WP_ = 160 MAm^−2^ does exceed 22T. Figure 8*b* shows the achievable toroidal magnetic field at the plasma geometric centre and shows that in comparison to the results of figure 3*b*, the field at the geometric centre at lower aspect ratios is more strongly influenced by the ability of the TF magnets to access *J*_WP_≥40 MAm^−2^ than by the ability to access $B_{\max}\geq 19\;T$.

**Figure 9.**
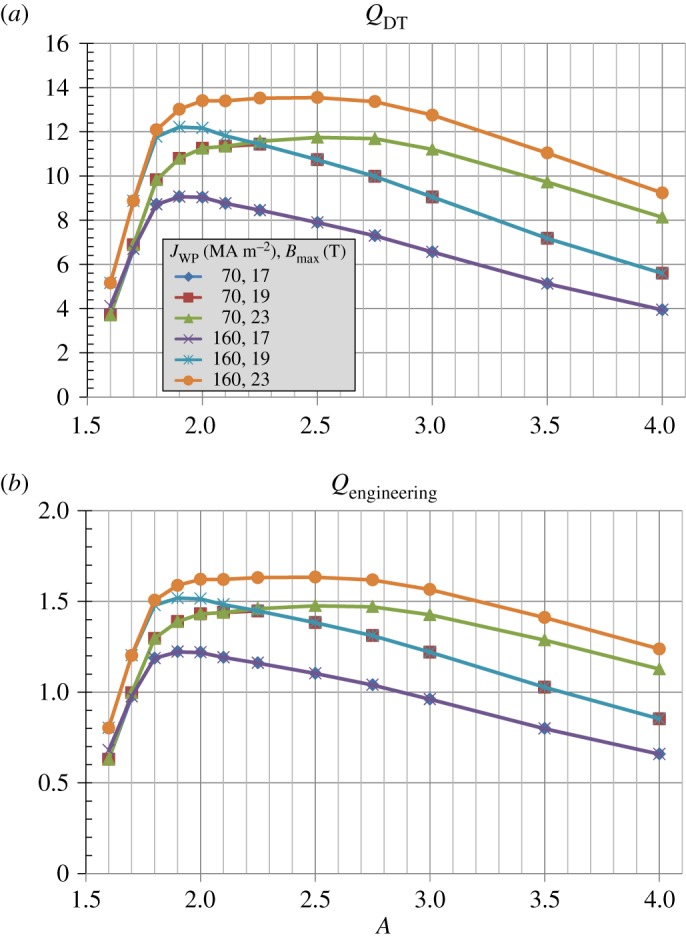
(*a*) Fusion gain, (*b*) engineering gain versus aspect ratio for various toroidal field coil maximum field and winding pack current density assumptions. (Online version in colour.)

**Figure 10.**
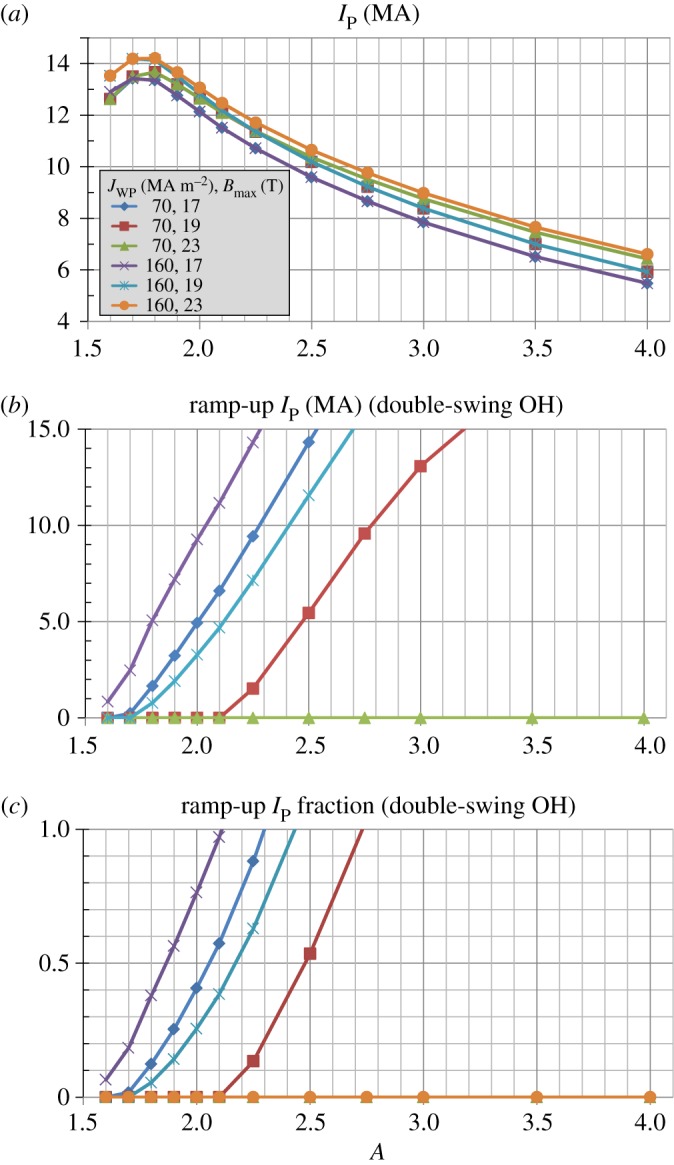
(*a*) Flat-top plasma current, (*b*) ramp-up plasma current for double-swing ohmic heating (OH) solenoid and (*c*) ramp-up plasma current fraction (relative to flat-top plasma current) for double-swing OH solenoid versus aspect ratio for various toroidal field coil maximum field and winding pack current density assumptions. (Online version in colour.)

**Figure 11.**
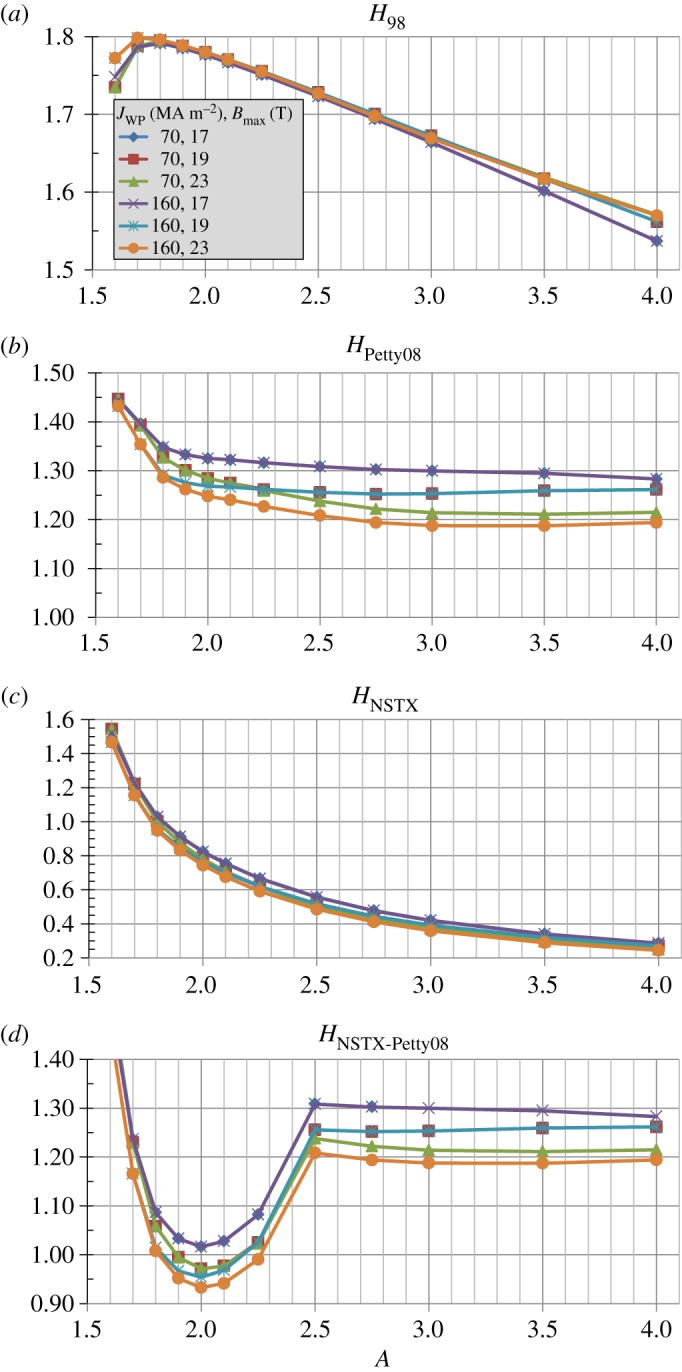
Normalized confinement enhancement factors for (*a*) ITER 98y2, (*b*) Petty08, (*c*) NSTX and (*d*) hybrid NSTX-Petty08 energy confinement scalings versus aspect ratio for various toroidal field coil maximum field and winding pack current density assumptions. (Online version in colour.)

For the same magnet parameters as figure 8, figure 9 shows that for $B_{\max}=19\;T$ the highest fusion and engineering gains occur near *A* = 2. However, as the maximum field is relaxed to the highest achievable (i.e. constrained by space for inboard TF coil structural support), there is a broad range of aspect ratio *A* = 2 to 3 with similar gains. Figure 10*a* shows that the plasma current versus aspect ratio depends relatively weakly on *J*_WP_ and $B_{\max}$ for the cases considered. However, figure 10*b* shows that the achievable ramp-up plasma current depends more strongly on the combination of *J*_WP_ and *B*_max_ with the lowest $B_{\max}$ and highest *J*_WP_ combination providing the most space for a central solenoid and highest inductive ramp-up plasma current. These calculations assume a double-swing OH solenoid is located inside the bore of the inner toroidal field coil support structure using a solenoid with 20T maximum field and *J*_WP_ = 70 MAm^−2^ [17]. Figure 10*c* shows the ratio of ramp-up plasma current to steady-state flat-top current is small or zero for *A* = 1.6 and can reach 1 for *A* as low as 2.1 for *J*_WP_ = 160 MAm^−2^ and $B_{\max}=17\;T$. As $B_{\max}$ is increased to 19 T, ramp-up current fractions above 1 are only possible for *A* = 2.4 to 2.7 depending on *J*_WP_. For $B_{\max}=23\;T$ all the inboard space is used for TF structural support and there is no space for a solenoid and the OH ramp-up current fraction is zero for all aspect ratios considered. Figure 11 shows the normalized confinement multiplier requirement trends versus aspect ratio are largely similar to those shown in figure 7, but figure 11*d* shows a clearer minimum H-factor requirement near 1 for *A* ≈ 2 for the hypothesized ad-hoc NSTX-Petty08 confinement scaling.

### (d) Fusion performance versus scaled normalized beta

**Figure 12.**
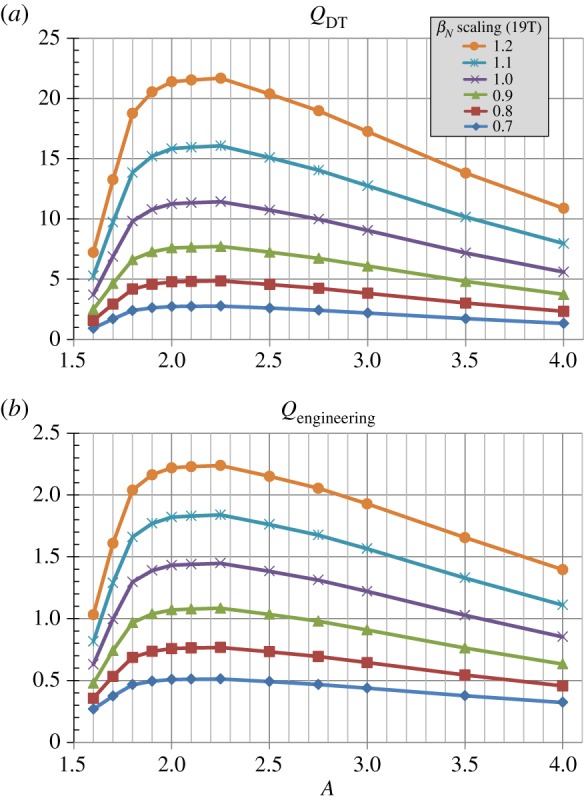
(*a*) Fusion gain, (*b*) engineering gain versus aspect ratio and total *β*_*N*_ scaled relative to the no-wall limit *β*_*N*_(*A*) from figure 1*b* for $B_{\max}=19\;T$ and *J*_WP_ = 70 MAm^−2^. (Online version in colour.)

**Figure 13.**
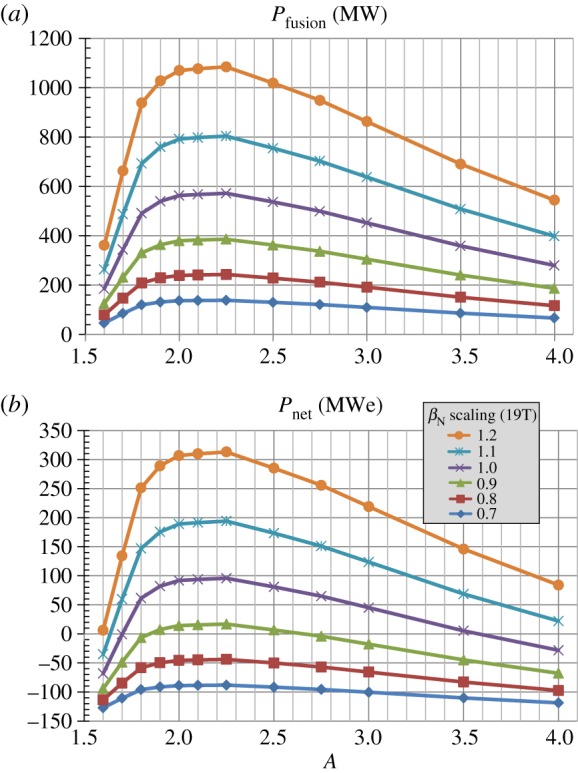
(*a*) Fusion power, (*b*) net electric power versus aspect ratio and total *β*_*N*_ scaled relative to the no-wall limit *β*_*N*_(*A*) from figure 1*b* for $B_{\max}=19\;T$ and *J*_WP_ = 70 MAm^−2^. (Online version in colour.)

As discussed in the introduction to §2, the strong dependence of the fusion power on *β*_*N*_ where *P*_fusion_∝*β*^4^_*N*_ is a strong motivation for accessing improved stability to pressure-driven modes for all aspect ratios. Figure 12 shows the fusion and engineering gains versus aspect ratio for a scan of total (thermal + fast-particle) *β*_*N*_ scaled by factors ranging from 0.7 to 1.2 × the *β*_*N*_(*A*) scaling of figure 1*b* assuming $B_{\max}=19\;T$ and *J*_WP_ = 70 MAm^−2^. As is evident from figure 12, both gains increase by approximately a factor of 8 as the no-wall limit *β*_*N*_ is scaled from 0.7 to 1.2 consistent with the *β*^4^_*N*_ scaling. Strictly speaking, the thermal *β*_*N*_ and bootstrap fraction should be considered in these scaling comparisons, but similar scaling trends are also observed when these parameters are considered. Figure 13 shows fusion power and net electric power for the same scan. Figure 13*a* shows that fusion powers exceeding 1 GW are projected for *β*_*N*_ values 20% above the assumed no-wall limit. Furthermore, figure 13*b* shows that net-electric powers above 200 MWe are projected for the same elevated *β*_*N*_ assumption. Thus, accessing plasma pressures above the no-wall stability limit relying on either passive/kinetic stabilization [47–58] and/or active RWM feedback control [59–62] can have a substantial impact on fusion performance and is potentially a requirement for modular approaches to steady-state power generation utilizing compact tokamaks [18].

**Figure 14.**
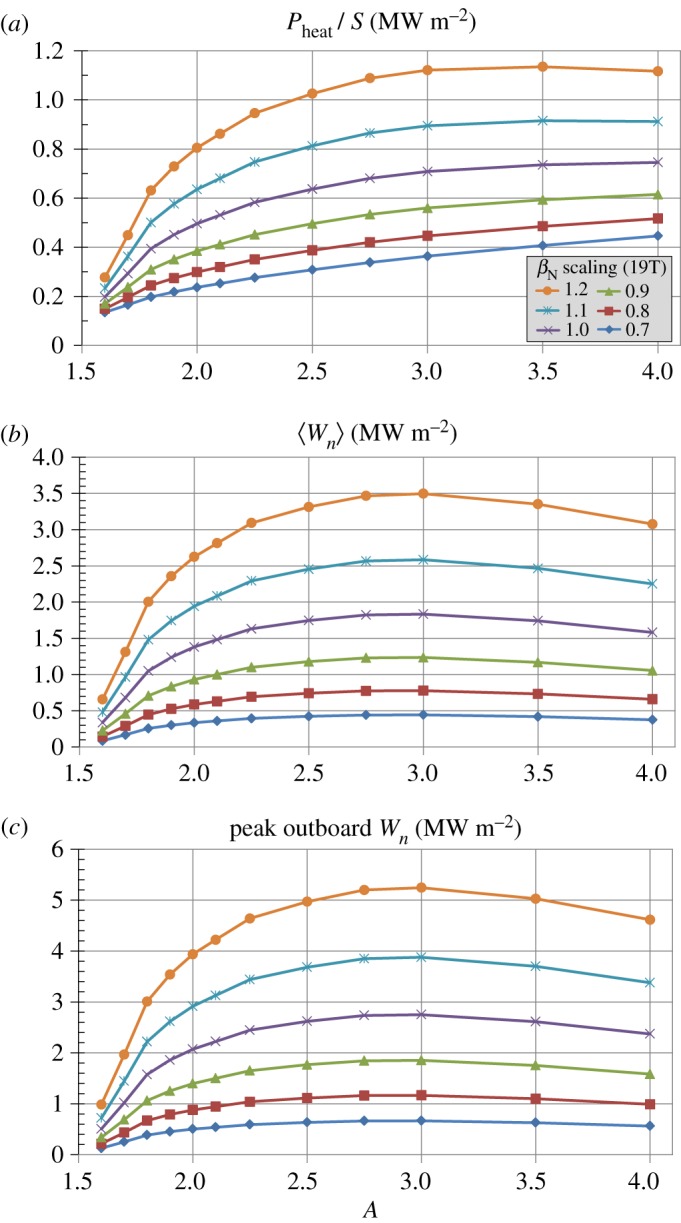
(*a*) Surface-average power exhaust flux, (*b*) surface-average neutron wall loading and (*c*) peak outboard neutron wall loading (all measured at the plasma surface) versus aspect ratio and total *β*_*N*_ scaled relative to the no-wall limit *β*_*N*_(*A*) from figure 1*b* for $B_{\max}=19\;T$ and *J*_WP_ = 70 MAm^−2^. (Online version in colour.)

Figure 14 shows that the power densities can be very high for these compact configurations operating at elevated *β*_*N*_. Figure 14*a* shows that the surface-average heat flux exceeds 1 MWm^−2^ for aspect ratios *A*≥2.5. Figure 14*b* shows that the surface-average neutron wall loading exceeds 3 MWm^−2^ for aspect ratios *A*≥2.2. Assuming an outboard mid-plane neutron wall loading peaking factor of 1.5 [23], figure 14*c* shows that the peak outboard neutron wall loading exceeds 5 MWm^−2^ for aspect ratios *A*≥2.5. Reducing the power exhaust and neutron wall loading parameters tends to favour lower aspect ratio due to larger plasma boundary (and first-wall) surface area when the plasma major radius is fixed.

**Figure 15.**
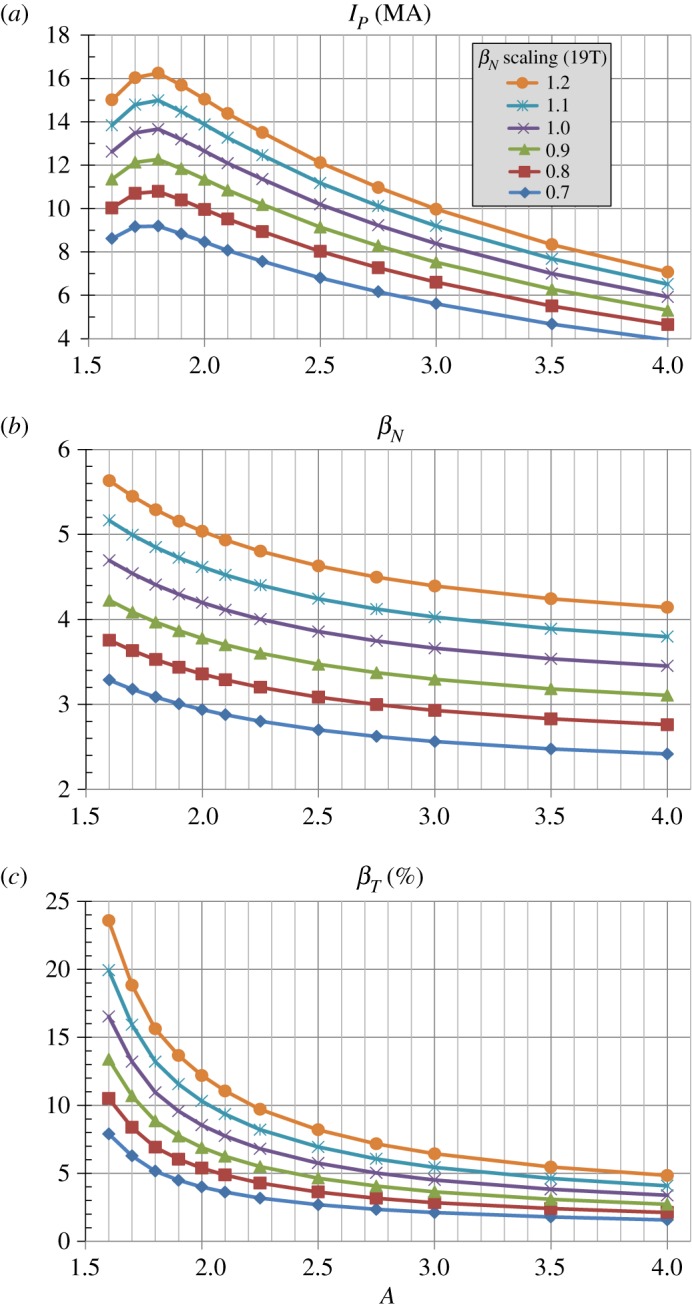
(*a*) Plasma current, (*b*) total normalized beta and (*c*) toroidal beta versus aspect ratio and total *β*_*N*_ scaled relative to the no-wall limit *β*_*N*_(*A*) from figure 1*b* for $B_{\max}=19\;T$ and *J*_WP_ = 70 MAm^−2^. (Online version in colour.)

**Figure 16.**
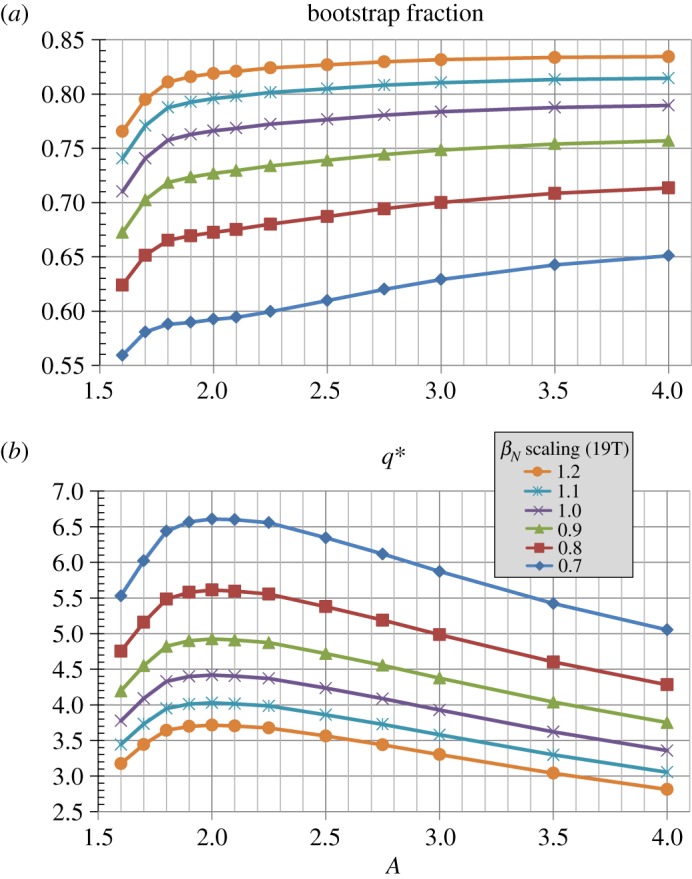
(*a*) Bootstrap fraction, (*b*) kink safety factor versus aspect ratio and total *β*_*N*_ scaled relative to the no-wall limit *β*_*N*_(*A*) from figure 1*b* for $B_{\max}=19\;T$ and *J*_WP_ = 70 MAm^−2^. (Online version in colour.)

Figure 15 shows trends for the plasma current and beta parameters for this normalized beta scan. Figure 15*a* shows that the plasma current scales approximately linearly with the scaled *β*_*N*_, and figure 15*b* shows the scaled *β*_*N*_ itself for reference. Since the toroidal beta is proportional to the normalized beta and plasma current, figure 15*c* shows the expected result that the toroidal beta *β*_*T*_ scales approximately quadratically with the scaled *β*_*N*_. Figure 16*a* shows that the bootstrap current fraction is highest at the highest *β*_*N*_ and is between 80 and 85% for nearly all aspect ratios considered. As the *β*_*N*_ is lowered, the bootstrap current (which is proportional to the thermal plasma pressure) also decreases and the non-inductive current drive from the 50 MW of 0.5 MeV NNBI contributes an increasingly larger fraction of the total current. Figure 16*b* shows that the highest *β*_*N*_ cases have the lowest kink safety factor q* consistent with these cases also having the highest plasma current. *A* ≈ 2 has the highest q* and nearly all cases have q*≥3 as a metric for stability against the external current-driven kink mode [63].

**Figure 17.**
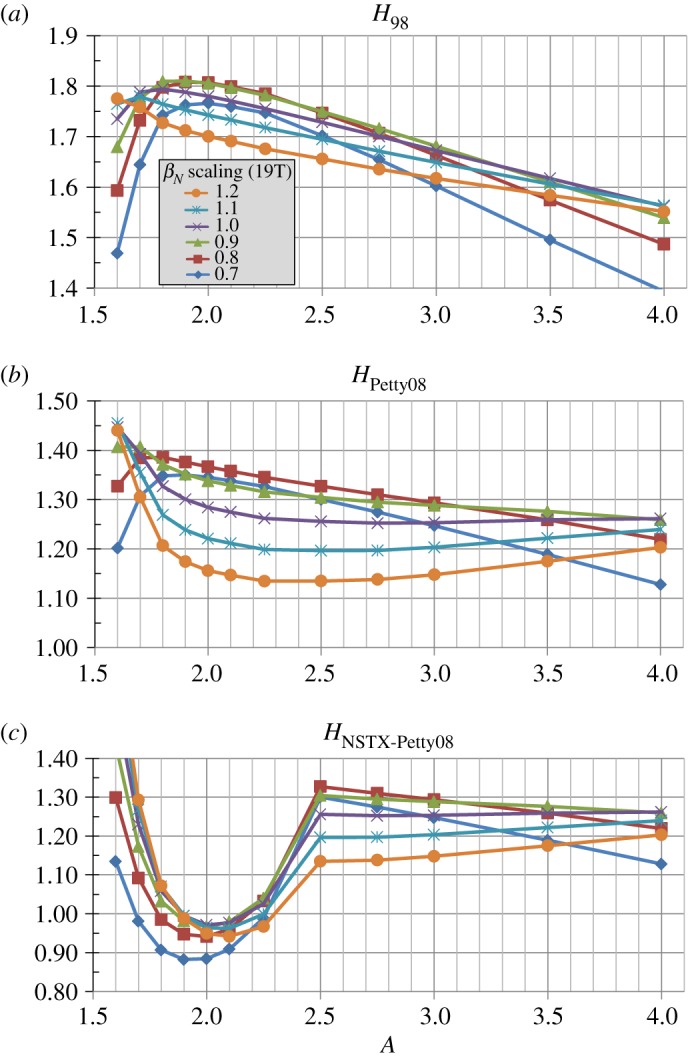
Normalized confinement enhancement factors for (*a*) ITER 98y2, (*b*) Petty08 and (*c*) hybrid NSTX-Petty08 energy confinement scalings versus aspect ratio and total *β*_*N*_ scaled relative to the no-wall limit *β*_*N*_(*A*) from figure 1b for $B_{\max}=19\;T$ and *J*_WP_ = 70 MAm^−2^. (Online version in colour.)

Figure 17 shows that for the set of confinement scalings treated here (ITER 98y2, Petty08, NSTX-Petty08) there is a non-monotic dependence on *β*_*N*_ values used. In particular, for the lowest values of *β*_*N*_(*A*) scaling factor considered (0.7 and 0.8), increasing *H* is required for increasing *β*_*N*_(*A*) scaling factor. However, as the scaling factor is further increased above values in the range of 0.9 to 1, the required *H* progressively decreases. This nonlinear result arises because the increased fusion power and alpha-driven plasma heating at higher assumed *β*_*N*_ scaling reduces the required confinement multiplier. Such results may point to the complexity of controlling potentially highly nonlinear plasma states in which increasing fusion gain increases the plasma pressure, bootstrap and total current, and thus the confinement time (through the increased plasma current) thereby further increasing the fusion gain until other effects intervene to saturate or limit the fusion gain. Such saturating effects include reduced fusion reactivity and/or increased plasma radiation power loss at very high plasma temperature, and limiting effects include pressure-driven disruptions.

### (e) Fusion performance sensitivity to confinement and stability limits

**Figure 18.**
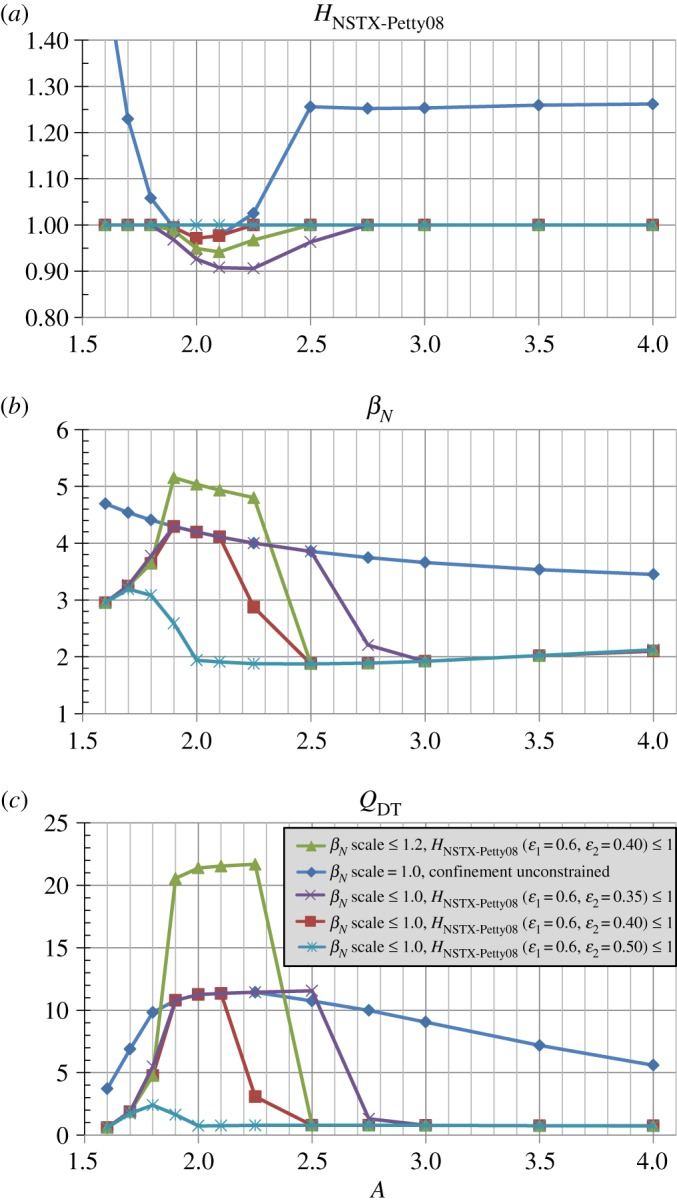
(*a*) Confinement multiplier, (*b*) total normalized beta and (*c*) fusion gain versus aspect ratio for a range of NSTX-Petty08 confinement model and *β*_*N*_ scaling assumptions for $B_{\max}=19\;T$ and *J*_WP_ = 70 MAm^−2^. (Online version in colour.)

The parametric scans addressed in previous sections dealt primarily with constraints on the maximum *β*_*N*_ and/or magnetic field or magnet current density while leaving the confinement unconstrained. To investigate the impact of constrained confinement using the NSTX-Petty08 model, figure 18 shows the confinement multiplier, achievable *β*_*N*_ and fusion gain assuming *H*_NSTX-Petty08_ ≤ 1 as shown in figure 18*a* (with the exception of a reference unconstrained case indicated by the blue diamond symbols). Figure 18*b* shows that the assumed *β*_*N*_ constraint is only accessible for narrow range of aspect ratios determined by the value of *ϵ*_2_ from the *ad hoc* NSTX-Petty08 confinement scaling model. In particular, as the aspect ratio for which ST confinement transitions to Petty08 confinement is reduced (i.e. *ϵ*_2_ is increased), an increasingly narrow window of sufficient confinement is accessible to reach the *β*_*N*_ constraint. If the transition aspect ratio is as low as *A* = 2 (i.e. *ϵ*_2_ is as large as 0.5) then no aspect ratio can reach the *β*_*N*_ constraint for *H*_NSTX−Petty08_ ≤ 1. It is also evident that for *H*_NSTX-Pett08_ ≤ 1 many aspect ratios have lower *β*_*N*_ ≈ 2 and much lower fusion gain *Q*_*DT*_ ≈ 1 than the *β*_*N*_ constraint would allow. This is due to the strong dependence of the gain on H as described in more detail in appendix A. These results also indicate that if ST scaling does in fact extend to lower collisionality and higher field and current in NSTX-U and MAST-U, important questions may still remain on how rapidly the transition from ST to conventional-A confinement occurs for intermediate *A* = 1.8 to 2.5.

**Figure 19.**
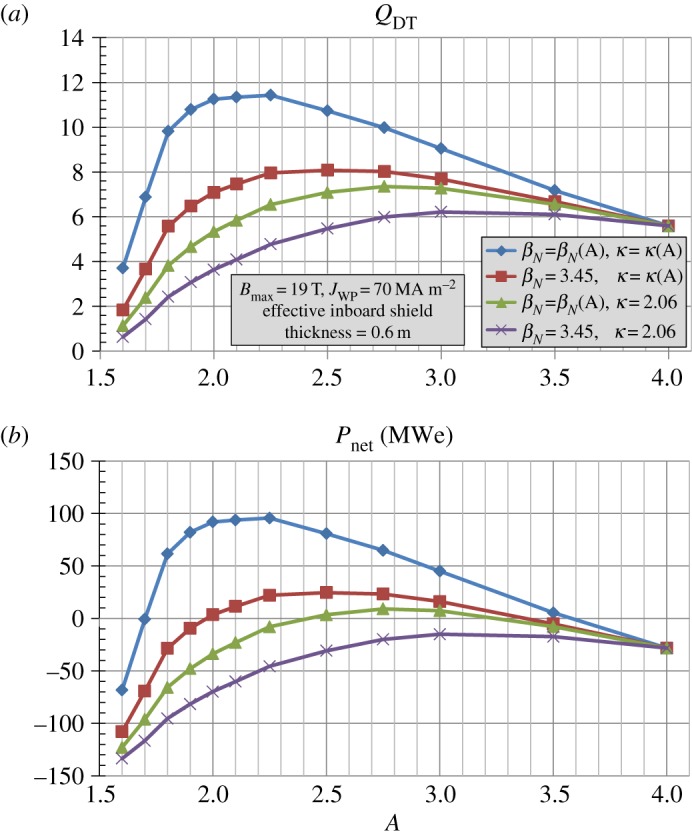
(*a*) Fusion gain *Q*_*DT*_, (*b*) net electrical power versus aspect ratio *A* for different assumptions for *β*_*N*_(*A*) and *κ*(*A*) at $B_{\max}=19\;T$ and *J*_WP_ = 70 MAm^−2^. (Online version in colour.)

Finally, figure 19 shows that for $B_{\max}=19\;T$ and *J*_WP_ = 70 MAm^−2^, the assumed aspect ratio dependence of *β*_*N*_ and *κ* has a significant impact on the achievable fusion gain and net electrical power. In particular, the fusion gain decreases by nearly a factor of 2 and net electrical power becomes negative if the *β*_*N*_ and *κ* are held constant at the values applicable to *A* = 4 (*β*_*N*_ = 3.45 and *κ* = 2.06). These results are similar to those shown in figure 2 indicating that the aspect ratio dependence of *β*_*N*_ and *κ* is important for a range of magnet parameters.

## 3. Conclusion

For tokamaks with plasma major radius as small as *R* = 3 m, net electric power production becomes accessible from a plasma stability standpoint if the toroidal field coil winding pack current density and maximum field at the coil are sufficiently high (17–20 T). As the maximum magnetic field at the TF coil is increased above approximately 19–20 T, magnet structural support requirements leave progressively less space available in the central bore until only small or no space remains for a central solenoid. In the limit of small or no central solenoid for plasma current formation, aspect ratios near 2 become increasingly attractive for maximizing fusion power and electrical power production. For such lower-*A* solutions to be viable, high toroidal field magnet current density is required and appears feasible if (for example) CORC HTS cables can be developed into large-bore TF magnets. Furthermore, the aspect ratio dependence of *β*_*N*_ and *κ* is important to include since such dependence can potentially increase the fusion gain by up to a factor of 2 relative to fixed *β*_*N*_ and *κ*. Lower-*A* configurations would require some fraction of the current ramp-up to be provided through non-inductive current overdrive or via other methods of current formation [64]. All of the compact tokamak scenarios studied here require elevated confinement relative to conventional aspect ratio scalings in order to access *n* = 1 no-wall beta values. Interestingly, for an *ad hoc* confinement scaling connecting the NSTX and Petty08 scalings, *A* = 2 − 2.3 (a range of aspect ratios not previously studied experimentally) lowers the required confinement multiplier to *H* ≈ 1. Operation near and above the *n* = 1 no-wall limit could be particularly advantageous since *P*_fusion_∝*β*^3−4^_*N*_ for high bootstrap current fraction scenarios for which kink safety factor q* ≥3 is intrinsically met and is not a stability constraint. Net electric powers of 200–300 MWe are projected at elevated *β*_*N*_ values ≈ 1.2 × the no-wall limit in a compact device with *R* = 3 m, but such configurations would have challenging exhaust heat fluxes and high neutron wall loading. These high power fluxes would lead to central HTS toroidal field magnet lifetimes of 1–2 full-power years from neutron irradiation damage potentially requiring blanket and central TF magnet change-out every 1–2 years if very high duty factor was ultimately achieved. HTS TF magnet lifetime could be increased by up to a factor of 5 by increasing the effective inboard shielding thickness from 60 cm to 70 cm [17]. Increased inboard shielding at fixed plasma major radius does however result in decreased plasma and engineering gains.

## Data accessibility

The digital data for this paper can be found at: https://dataspace.princeton.edu/jspui/handle/88435/dsp010v838334m.

## Appendix A. Fusion gain scaling for steady-state tokamaks

In this Appendix, fusion gain scalings are derived for high-field steady-state tokamaks with high-bootstrap-fraction configurations for which limits on the external current drive are typically more constraining than kink stability limits on the minimum edge safety factor. Such scalings can be expressed as a combination of both dimensional and dimensionless parameters most relevant to reactor design to aid understanding of the trade-offs between different parameters.

The deuterium-tritium (D-T) fusion power *P*_f_ in a magnetic confinement system scales as *P*_f_∝*n*^2^〈*σv*〉_*DT*_*V* [65] which for ion temperatures in the range of 8-30keV is approximately proportional to *n*^2^*T*^2^*V* ∝*p*^2^*V* = *β*^2^_*T*_*B*^4^_*T*0_*V* where *β*_*T*_≡2*μ*_0_〈*p*〉/*B*^2^_*T*0_, 〈*p*〉 is the volume-averaged plasma pressure, *B*_*T*0_ is the vacuum toroidal field at the plasma geometric centre and *V* is the plasma volume. The maximum *β*_*T*_ is limited by plasma magnetohydrodynamic instabilities and the maximum toroidal magnetic field in the plasma is limited by engineering constraints including maximum field, force, current density and/or cooling of the magnet. Furthermore, *p*∝*P*_*h*_*τ*_*E*_/*V* where *P*_*h*_ is the plasma heating (loss) power and *τ*_*E*_ is the energy confinement time. For the scalings here, core radiation losses and divertor power exhaust challenges are not considered and are topics for future study. The energy confinement time can be expressed as a scaling in terms of dimensional parameters as *τ*_*E*_∝*HI*^*α*_*I*_^_*P*_*B*^*α*_*B*_^_*T*_*n*^*α*_*n*_^_*e*_*P*^−*α*_*P*_^_*h*_*R*^*α*_*R*_^*κ*^*α*_*κ*_^*ϵ*^*α*_*ϵ*_^ where *H* is the confinement multiplier, *I*_*P*_ is the plasma current, *n*_*e*_ is the plasma electron density, *R* is the plasma major radius, *κ* is the plasma boundary elongation and *ϵ* is the plasma inverse aspect ratio, where *ϵ*≡*A*^−1^ and *A*≡*R*/*a* and *a* is the plasma minor radius. From these definitions is also follows that *V* ∝*κRa*^2^∝*κR*^3^*ϵ*^2^. The fusion power therefore scales as *P*_f_∝*H*^2^*I*^2*α*_*I*_^_*P*_*B*^2*α*_*B*_^_*T*_*n*^2*α*_*n*_^_*e*_*P*^2(1−*α*_*P*_)^_*h*_*R*^2*α*_*R*_−3^*κ*^2*α*_*κ*_−1^*ϵ*^2*α*_*ϵ*_−2^.

The plasma heating power is the sum of the auxiliary heating and current drive power *P*_aux_ and the self-heating power from alpha-particles *P*_*α*_ = *λ*_*DT*_*P*_f_: *P*_*h*_ = *P*_aux_ + *λ*_*DT*_*P*_f_ = *P*_aux_(1 + *λ*_*DT*_*Q*_*DT*_) where *Q*_*DT*_≡*P*_f_/*P*_aux_ and *λ*_*DT*_ ≈ 0.2, i.e. 20% of the DT fusion power is in confined *α* particles that can heat the plasma. Thus, *P*_f_ = *Q*_*DT*_*P*_aux_ and *Q*_*DT*_ are determined from the solution of the equation *Q*_*DT*_/(1 + *λ*_*DT*_*Q*_*DT*_)^2(1−*α*_*P*_)^ = *Q*^*^_*DT*_ where *Q*^*^_*DT*_∝*H*^2^*I*^2*α*_*I*_^_*P*_*B*^2*α*_*B*_^_*T*_*n*^2*α*_*n*_^_*e*_*P*^2(1−*α*_*P*_)−1^_aux_*R*^2*α*_*R*_−3^*κ*^2*α*_*κ*_−1^*ϵ*^2*α*_*ϵ*_−2^. It is noted that for *λ*_*DT*_*Q*_*DT*_≪1 it follows that *Q*_*DT*_ ≈ *Q*^*^_*DT*_. For *λ*_*DT*_*Q*_*DT*_≫1 it follows that $Q_{DT}\propto {\left(Q_{DT}^{*}\right)}^{\frac{1}{2\alpha_{P}-1}}$ which is a sensitive function of *α*_*P*_ and implies *α*_*P*_ > 0.5 is required for physically meaningful results using this simple zero-dimensional scaling. Thus, for typical values of *α*_*P*_ ≈ 0.7 and all other parameters held fixed, *P*_f_∝*H*^2^ at low fusion gain and *P*_f_∝*H*^5^ at high fusion gain indicating the fusion power is a very strong function of the confinement multiplier as the plasma transitions from the burning plasma regime (*Q*_*DT*_ = 5-10) towards ignition.

The density and MHD pressure limits are important parameters for reactor design. The empirical density limit for tokamaks [66,67] can be expressed as Greenwald density fraction $f_{\mathrm{gw}}\equiv {\bar{n}}_{e}/n_{\mathrm{gw}}\leq 1$ where *n*_gw_[10^20^ m^−3^]≡*I*_*P*_[*MA*]/*πa*^2^ and thus *n*_*e*_∝*f*_gw_*I*_*P*_/*a*^2^. From this scaling it follows that the normalized fusion gain scales as:

A 1 $$Q_{DT}^{*}\propto H^{2}I_{P}^{2(\alpha_{I}+\alpha_{n})}B_{T}^{2\alpha_{B}}f_{\mathrm{gw}}^{2\alpha_{n}}P_{\mathrm{aux}}^{2(1-\alpha_{P})-1}R^{2\alpha_{R}-3-4\alpha_{n}}\kappa^{2\alpha_{\kappa }-1}\epsilon^{2\alpha_{\epsilon }-2-4\alpha_{n}.}$$

Similarly, the plasma current *I*_*P*_ can be recast as *I*_*P*_ = (*I*_*P*_/*aB*_*T*_)*aB*_*T*_ and since *I*_*P*_/*aB*_*T*_∝*β*_*T*_/*β*_*N*_ then *I*_*P*_∝(*β*_*T*_/*β*_*N*_)*aB*_*T*_. From this relation it follows that:

A 2 $$Q_{DT}^{*}\propto {\left(\frac{\beta_{T}}{\beta_{N}}\right)}^{2(\alpha_{I}+\alpha_{n})}B_{T}^{2(\alpha_{B}+\alpha_{I}+\alpha_{n})}R^{2(\alpha_{R}+\alpha_{I}-\alpha_{n})-3}\epsilon^{2(\alpha_{\epsilon }+\alpha_{I}-\alpha_{n}-1)}.$$

Since *β*_*T*_ = *ϵ*^1/2^(*C*_*BS*_/*f*_*BS*_)((1 + *κ*^2^)/2)(*β*_*N*_/2)^2^ [63,68,69] and one can for simplicity approximate (1 + *κ*^2^)/2 ≈ 0.9*κ*^3/2^ for *κ* values of interest (*κ* = 1.5 − 3), it follows that *β*_*T*_/*β*_*N*_∝((*C*_*BS*_/*f*_*BS*_)*β*_*N*_)*κ*^3/2^*ϵ*^1/2^. Combining all these scalings results in the following scaling for *Q*^*^_*DT*_ :

A 3 $$Q_{DT}^{*}\propto H^{2}{\left(\frac{\beta_{N}C_{BS}}{f_{BS}}\right)}^{c_{\beta }}B_{T}^{c_{B}}f_{\mathrm{gw}}^{c_{\mathrm{gw}}}P_{\mathrm{aux}}^{c_{P}}R^{c_{R}}\kappa^{c_{\kappa }}\epsilon^{c_{\epsilon }}$$

A 4 $$c_{\beta }=2(\alpha_{I}+\alpha_{n})=+2.68\;[98y2]=+2.14\;\left[\mathrm{Petty}08\right]$$

A 5 $$c_{B}=2(\alpha_{B}+\alpha_{I}+\alpha_{n})=+2.98\;[98y2]=+2.74\;\left[\mathrm{Petty}08\right]$$

A 6 $$c_{gw}=2\alpha_{n}=+0.82\;[98y2]=+0.64\;\left[\mathrm{Petty}08\right]$$

A 7 $$c_{P}=1-2\alpha_{P}=-0.38\;[98y2]=+0.06\;\left[\mathrm{Petty}08\right]$$

A 8 $$c_{R}=2(\alpha_{R}+\alpha_{I}-\alpha_{n})-3=+1.98\;[98y2]=+2.04\;\left[\mathrm{Petty}08\right]$$

A 9 $$c_{\kappa }=3\alpha_{\kappa }+2(\alpha_{I}+\alpha_{n})-1=+4.02\;[98y2]=+3.78\;\left[\mathrm{Petty}08\right]$$

A 10 $$\text{and}\;c_{\epsilon }=2\alpha_{\epsilon }+3\alpha_{I}-\alpha_{n}-2=+1.54\;[98y2]=+1.61\;[\mathrm{Petty}08].$$

Assuming the Petty08 scaling is more representative of compact steady-state tokamak pilot-plant confinement, it is evident that *Q*^*^_*DT*_ scales approximately as:

A 11 $$Q_{DT}^{*}\propto f_{gw}^{2/3}\epsilon^{5/3}{\left(\frac{HR\beta_{N}(\epsilon )C_{BS}}{f_{BS}}\right)}^{2}B_{T}{\left(\epsilon \right)}^{3}\kappa {\left(\epsilon \right)}^{4}$$

and that the aspect ratio dependence of *β*_*N*_, *B*_*T*_ and *κ* are important factors in determining the optimal aspect ratio for maximizing fusion gain when other parameters are held fixed.
